# The effect of COVID-19 pandemic on admissions for cannabis-induced psychotic disorder

**DOI:** 10.1192/j.eurpsy.2022.2051

**Published:** 2022-09-01

**Authors:** A. Elias De Sousa, A.S. Machado, F. Andrade, M. Roque Gonçalves, M. Vieira-Coelho

**Affiliations:** 1 Centro Hospitalar Universitário de São João, Serviço De Psiquiatria, Porto, Portugal; 2 FMUP, Departamento De Neurociências Clínicas E Saúde Mental, Porto, Portugal

**Keywords:** Cannabis-induced psychosis, drugs, Cannabis, pandemic

## Abstract

**Introduction:**

Cannabis-induced psychotic disorder (CIPD) is defined by the development of psychotic symptoms during or briefly after intoxication with cannabis or withdrawal from cannabis. The social measures and restrictions implemented following the COVID-19 pandemic might have had an impact on cannabis availability, as suggested by patients from our clinical practice, reporting a shortage of the substance.

**Objectives:**

To compare sociodemographic, clinical characteristics and admission rates of inpatient treatment for cannabis-induced psychotic disorder in COVID-19 pandemic period and pre-pandemic period.

**Methods:**

Retrospective observational study of inpatient admissions for CIPD in a psychiatry inpatient unit of a tertiary hospital. The statistical analysis was performed using SPSS software, version 27.0.

**Results:**

Our sample included 120 inpatient admissions, corresponding to 80 patients. Compared to 2018 and 2019, in 2020 there was an overall reduction of 21.5% in inpatient admissions (n=618, 549 and 458, respectively). The number of admissions for CIPD in 2018, 2019, 2020 and 2021 up to september were, respectively, 29, 32, 10 and 31 (5.2%, 6.1%, 2.2% and 7.2% of respective annual admissions). We found no statistically significant differences regarding sociodemographic and clinical characteristics in patients admitted for CIPD during 2020.

**Conclusions:**

These results suggest a disproportionate reduction of inpatient admissions due to CIPD in 2020, followed by an expressive increase in the number of admissions in 2021up to september. This might be related to cannabis availability returning to regular levels. However other factors must be considered, such as the delay of treatment due to reduced accessibility to health care.

**Disclosure:**

No significant relationships.

